# Acetylation & Co: an expanding repertoire of histone acylations regulates chromatin and transcription

**DOI:** 10.1042/EBC20180061

**Published:** 2019-04-02

**Authors:** Claire E. Barnes, David M. English, Shaun M. Cowley

**Affiliations:** Department of Molecular and Cell Biology, University of Leicester, Leicester, U.K.

**Keywords:** Acetylation, Chromatin, Histone, Transcription

## Abstract

Packaging the long and fragile genomes of eukaryotic species into nucleosomes is all well and good, but how do cells gain access to the DNA again after it has been bundled away? The solution, in every species from yeast to man, is to post-translationally modify histones, altering their chemical properties to either relax the chromatin, label it for remodelling or make it more compact still. Histones are subject to a myriad of modifications: acetylation, methylation, phosphorylation, ubiquitination etc. This review focuses on histone acylations, a diverse group of modifications which occur on the ε-amino group of Lysine residues and includes the well-characterised Lysine acetylation. Over the last 50 years, histone acetylation has been extensively characterised, with the discovery of histone acetyltransferases (HATs) and histone deacetylases (HDACs), and global mapping experiments, revealing an association of hyperacetylated histones with accessible, transcriptionally active chromatin. More recently, there has been an explosion in the number of unique short chain ‘acylations’ identified by MS, including: propionylation, butyrylation, crotonylation, succinylation, malonylation and 2-hydroxyisobutyrylation. These novel modifications add a range of chemical environments to histones, and similar to acetylation, appear to accumulate at transcriptional start sites and correlate with gene activity.

## Histone modification: crowbar and post-it note

Packaging DNA into nucleosomes helps protect the long fragile genomes of eukaryotic species. However, in doing so it presents a constant physical barrier to the protein machinery required for its replication, repair and transcription. Wrapped up tightly in its histone overcoat, how are cells able to gain access to the underlying DNA? Universally, in species as diverse as brewer’s yeast, fruit flies, worms and man, the answer is to chemically modify the histones to either help open up or compress the chromatin still further. And what a variety of modifications there are: acetylation, methylation, phosphorylation, ubiquitination, sumoylation, adenylation etc. So many that we would probably need another issue of *Essays in Biochemistry* to fit them all in and do them justice. This review will therefore focus on acetylation and a range of newly identified ‘acylations’ while recommending the reader to a number of other reviews which comprehensively cover these additional modifications [1–4].

**Figure 1:**
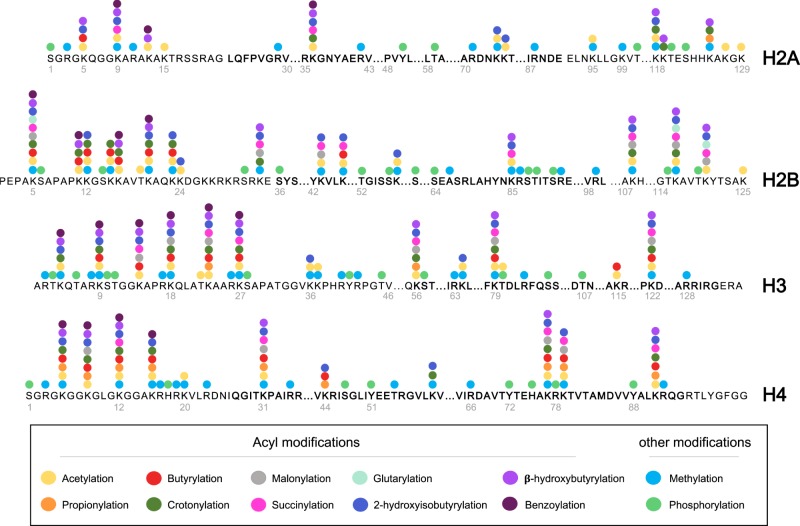
Schematic representation of histone modifications Identified sites of histone acylation, methylation and phosphorylation in the four core histones are indicated. Amino acids located in the globular histone domains are shown in bold. Based on PTMs identified in [83,84,111].

Acylation (which includes acetylation) occurs on Lysine residues through the addition of an acyl group from an acyl-CoA donor to the ε-amino group of the Lysine side chain. Each of the core histones contains a globular core domain and a flexible N-terminal tail with an array of highly conserved Lysine residues, which being positively charged, have a natural affinity for both the DNA backbone and a negatively charged patch on neighbouring nucleosomes. The addition of the acyl group masks the positive charge on the Lysine residue, thereby reducing the affinity of the tail for chromatin, leaving the underlying DNA more exposed. This mechanism is amplified by the number of Lysine residues present in each N-terminal tail: 4 of the first 15 residues in histone H2A are Lysine (27%), 8 of 24 in H2B (33%), 8 of 36 in H3 (22%) and 5 of 20 in H4 (20%) (Figure 1). The nucleosome has two copies of each core histone so that the 146 bp of DNA is surrounded by a flexible Lysine-rich environment, ripe for modification. It is worth bearing in mind that Lysine residues can also be methylated and ubiquitinated, which are two of the most abundant histone modifications. There is, therefore, a direct competition between different chemical modifications (Figure 1). For example, if H3K27 is acetylated (H3K27ac – a marker of open chromatin), then it cannot be methylated (H3K27me3 – a marker of repressed chromatin), resulting in opposing transcriptional readouts [5]. Lysine acetylation plays a second role, in addition to changing the charge on the histone tail, it also functions as a binding site for proteins bearing a bromodomain (BD), which tend to be proteins with a pro-transcriptional function (discussed in detail below). Together, these activities promote a more open, less condensed form of chromatin that is transcriptionally permissive. Indeed, genes whose underlying chromatin is acylated are more likely to be transcribed [6–11].

Histone acetylation is often referred to as an ‘epigenetic’ modification, that is, a code which sits on top of the genetic code, regulating access to the DNA. However, there is much debate as to whether histone modifications are really epigenetic or not, since this characterisation implies at least a degree of heritability, i.e. that sites of histone acetylation are passed on from mother cell to daughter cell following division. Despite much effort in characterising the proteins which regulate acylation levels in cells, a mechanism for the stable inheritance of histone acetylation has remained elusive, suggesting that epigenetic is not quite the right term. Another argument against is that the half-life of acetyl-lysine (K_Ac_) is typically 30 min to 2 h [12,13], so that if it does represent a code then it is a short-lived one. K_Ac_ is a highly dynamic modification being constantly added and removed to enable DNA accessibility when required. Indeed, there is evidence to suggest that cycles of acetylation and deacetylation are required for active gene transcription [14–16]. One way to view histone acylation is as part of a broad signalling mechanism [17], part crowbar (physically opening chromatin) and part Post-it Note (a temporary reminder that a job needs doing here), allowing DNA binding factors the access needed to get on with their job.

## Histone acetylation promotes open chromatin and gene activation

Acetylation was the first identified histone acyl modification and is the most prevalent [18,19]. With remarkable prescience, Allfrey et al. [18] not only associated acetylation with facilitating RNA synthesis from nucleosomal DNA, but also hypothesised that histone acetylation was a mechanism for the dynamic regulation of gene transcription *in vivo*. Since then the association of increased levels of histone acetylation with transcriptional activation has been demonstrated by numerous studies [6,20,21]. Evidence for histone acetylation preceding transcription has been shown through reports of global increases in acetylation occurring prior to global increases in mRNA [20] and the association of acetylation at inducible gene promoters prior to their stimulation [21]. Histone acetylation as a potential causal agent for transcriptional activation was further substantiated by the identification of enzymes capable of catalysing the addition of and removal of acetyl groups, histone acetyltransferases (HATs) and histone deacetylases (HDACs) respectively [22–24]. The previous association of HATs and HDACs as transcriptional regulators further cemented the functional association of histone acetylation with transcription [25,26]. However, a word of caution, transcriptional regulation is more complex than acetylation = on and deacetylation = off, as several studies have indicated a requirement for HDAC activity in transcriptional activation [14,16,27–29]. The function of HATs and HDACs in the regulation of transcription is further complicated by their ever-expanding activities towards non-histone substrates as well as histones [12,30,31]. It may therefore be more apt to call these enzymes lysine acetyltransferases (KATs) and lysine deacetylases (KDACs) respectively, however for continuity with other literature we will continue to use HATs and HDACs throughout this review.

Within the context of chromatin, hyperacetylation of histone tails reduces the thermal stability of nucleosomes and a H4 histone tail–DNA complex [32,33], as well as increasing susceptibility to DNase I digestion [34–36]. High levels of histone acetylation have also been associated with a reduction in the formation of higher order or compacted chromatin structures [37,38], with H4K16ac having been linked to a failure of the formation of the 30-nm chromatin fibre and associated with transcriptionally active chromatin fractions [39]. The crystal structure of a nucleosome indicates an interaction occurs between H4K16 and a highly acidic patch on the H2A/H2B dimer of adjacent nucleosomes [40]. Modelling of the interaction of the H4 tail with adjacent nucleosomes has suggested the acetylation of H4K16 impairs and weakens the internucleosomal interaction of the H4 tail with the acidic H2A/H2B patch [41]. Acetylation also occurs within the core globular domains of histones (see Figure 1) [42,43]. A number of these modifications are situated at histone–DNA interacting regions and have the potential to modulate their interaction [44,45]. Indeed, alteration of DNA–histone interactions has been demonstrated, with acetylation of K115, K122 and K64 reducing DNA-binding affinity and increasing nucleosome mobility [46,47].

## Site-specific lysine acetylation regulates gene expression

A plethora of specific K_Ac_ sites have been identified in each of the core histones (aided by high-resolution MS) that occur predominantly in the N-terminal tails (shown in Figure 1) [42,48,49]. A key development to investigating their function has been the generation of a range of antibodies which recognise specific histone K_Ac_ modifications [50]. In particular, they have allowed ChIP followed by next-generation sequencing (ChIP-seq) studies to map individual K_Ac_ sites across the genome, revealing histone acetylation in the proximity (1–2 kb) of transcription start sites (TSS) of actively transcribed genes [7–9]. However, there is some variation in the localisation, with H3K9ac and H3K27ac highly correlated to the TSS of active genes, whereas H4K12ac and H4K16ac are present at both TSS and along the gene body [8]. Distal regulatory regions (e.g. enhancers) have also been correlated with increased levels of H3K27ac [51,52] and localisation of the HAT, p300, in combination with mono-methylation of H3K4 [51,53,54]. However, the presence of specific histone modifications at enhancers is more complex, as recently H3K4 mono-, di- and tri-methylation have all been identified at enhancers and shown to correlate with enhancer RNA (eRNA) transcript levels (with H3K4me3 at sites of highest eRNA transcription) [55]. Furthermore, H3K16ac and the acetylation of globular domain residues H3K122 and H3K64 have also been associated with enhancers, which often lack H3K27ac (a classical mark of active enhancers) [47,56,57].

Mammalian cells contain numerous gene copies for each of the core histones [58] making mutagenesis studies of individual Lysine residues technically problematic. However, mutational analyses in yeast histone H4 demonstrated that Lys→Arg mutations at positions H4K5, K8 and K12 had additive effects upon gene expression changes, whereas K16R showed a greater individual effect, indicating a functionally distinct role for H4K16ac at least at a subset of genes [59]. Studies in mammalian systems have largely utilised *in vitro* transcriptional assays with recombinant nucleosomes to examine the function of specific histone acetylation modifications in gene regulation. For example, H3K14ac has been shown to be required for promoter nucleosome disassembly through Nap1 with an acetyl blocking H3K14R mutant preventing transcription and nucleosome eviction [60]. Recently, H3K9ac has been linked to the recruitment of the super elongation complex (SEC) to promote RNA pol II pause release, with mutation of Lysine to Arginine resulting in decreased transcription (due to increased RNA pol II pausing) [61]. The acetylation of globular domain residues, H3K56, H3K115 and H3K122, was investigated in *Drosophila* through acetyl mimicking (Gln) or acetyl blocking (Arg), mutations and demonstrated varied effects upon development, indicating potentially distinct functions of these modifications [62]. The acetylation of globular domain residues H3K122 and H3K64 has also been studied in mammalian systems, with overexpression of the acetyl mimicking H3K64Q resulting in increased gene expression and H3K122Q showing increased transcriptional activation in *in vitro* transcriptional assays [47,63]. The mechanism by which acetylation of H3K122 and H3K64 are proposed to increase transcription levels is by reducing DNA–histone interactions, which results in increased eviction of nucleosomes from the DNA [47,63].

## Reading the runes: recognising and deciphering the pattern of histone acylations

Recognising the 50+ specific sites of histone acetylation (Figure 1) is essential for the propagation of the ‘signal’ to downstream processes and functions. The major protein domain associated with K_Ac_ binding is the BD, although two other domains, the double PHD finger domain and the YEATS domain, are also capable of recognising K_Ac_ residues on histones [64–67]. In mammalian species approximately 61 BDs in 46 proteins have been identified, which include histone modifying enzymes and chromatin remodelling complexes, further indicating the close association between K_Ac_ recognition and chromatin state. A non-specific DNA binding capacity has also been identified in several BDs indicating a potential mechanism for enhancing and stabilising BD–chromatin interactions [68,69]. BDs are also implicated in additional functions such as non-histone K_Ac_ recognition. BRD3 for example, has been shown to bind to an acetylated form of the transcription factor, Gata1 [70]; and the second BD of BRD4 has the capacity to interact with acetylated cyclin T1 (a core component of P-TEFb), although this interaction alone is not sufficient for full activation of P-TEFb dependent transcription [71]. Inhibition of the p300/CBP BD caused no overall change in the localisation of p300, but reduced the levels of H3K27ac at enhancers indicating a role for the BD in regulating the catalytic activity towards H3K27 [72]. A key concept, highlighted throughout the study of chromatin PTMs, is the high degree of cross-talk between different modifications. For example, the tandem Tudor domain of SGF29 is essential for the recruitment of the SAGA HAT complex to sites of H3K4me3, which enables the processive acetylation of H3 tails [73,74]. Phosphorylation of H3S10 promotes GCN5-mediated acetylation of H3K14 through enhanced binding of GCN5 to the H3 tail [75,76]. H3S10 phosphorylation has also been shown to recruit the HAT, MOF, via the adaptor protein 14-3-3 and this phosphorylation-dependent recruitment is required for the acetylation of H4K16 [77].

## A constellation of novel acylations

As discussed above, histone acetylation was discovered in the 1960s and has been characterised extensively over the last 50 years with the discovery of HATs and HDACs, and the global mapping of these modifications across the genome. Indeed it would have been reasonable to conclude *that’s all she wrote* in relation to histone acylation. However, over the last few years there has been an explosion in the number of unique short-chain histone Lysine ‘acylations’ identified by MS, these include: propionylation (K_Pr_) [78], butyrylation (K_Bu_) [78], crotonylation (K_Cr_) [42], succinylation (K_Succ_) [79], malonylation (K_Mal_) [79], 2-hydroxyisobutyrylation (K_Hib_) [80], glutarylation (K_Glu_) [81], β-hydroxybutyrylation (K_Bhb_) [82], and most recently benzoylation (K_Bz_) [83] (summarised in Table 1). These modifications arise from their corresponding acyl-CoAs (e.g. propionyl-CoA, crotonyl-CoA etc.) and have different chemical properties. Hydrophobic groups (K_Pr_, K_Bu_, K_Cr_ and K_Bz_) neutralise the positive charge of lysine residues (like acetylation), the acidic groups (K_Succ_, K_Mal_ and K_Glu_) change the positive charge to a negative charge, while polar groups (K_Hib_ and K_Bhb_) allow hydrogen bond formation with interacting molecules. K_Bz_ stands out as the only known histone PTM with an aromatic acyl group, while K_Cr_ is planar and K_Bhb_ and K_Hib_ are branched (for a more comprehensive review of histone acylations, see [84]). An initial question to arise from the discovery of these modifications is whether they are ‘written’ and ‘erased’ by the same HATs and HDACs that control acetylation. A variety of *in vivo* and *in vitro* studies have demonstrated that the known HAT and HDAC families have wide-ranging acylation and deacylation capabilities (summarised in Table 1). The results of these studies suggest that HATs/HDACs show limited specificity for acylations, exemplified by the wide range of p300/CBP activities [10,85]. To date, no enzymes in addition to HATs and HDACs have been shown to be responsible for directly adding or removing these acylations. However, other enzymes do play roles in regulating their levels, for example the α-KGDH complex increases the local concentration of succinyl-CoA allowing GCN5 to succinylate H3K79 [86]. While CDYL acts as crotonyl-CoA hydratase, negatively regulating histone K_Cr_ [87], indicating further complexity in the regulation of histone acylations.

**Table 1:** Summary of alternative histone acyl modifications

Acyl group structure	Properties	HATs	HDACs
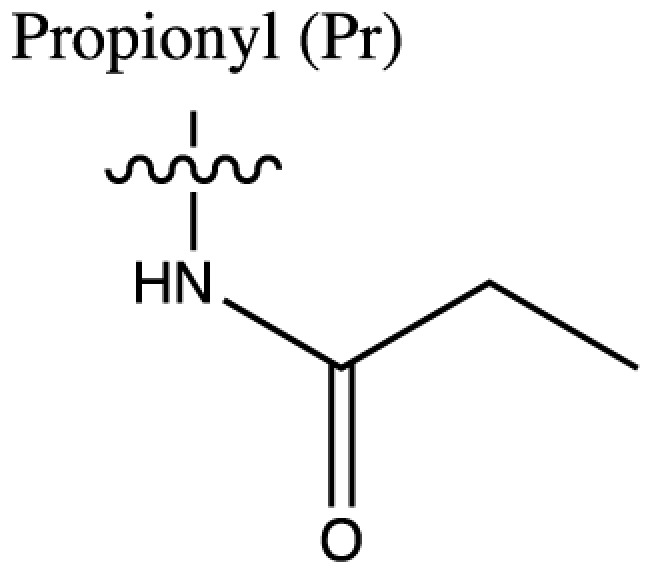	Hydrophobic	p300/CBP [79,93], PCAF [112], GCN5 [93,113], MOF, HBO1, MOZ [114]	SIRT1/2/3 [115]
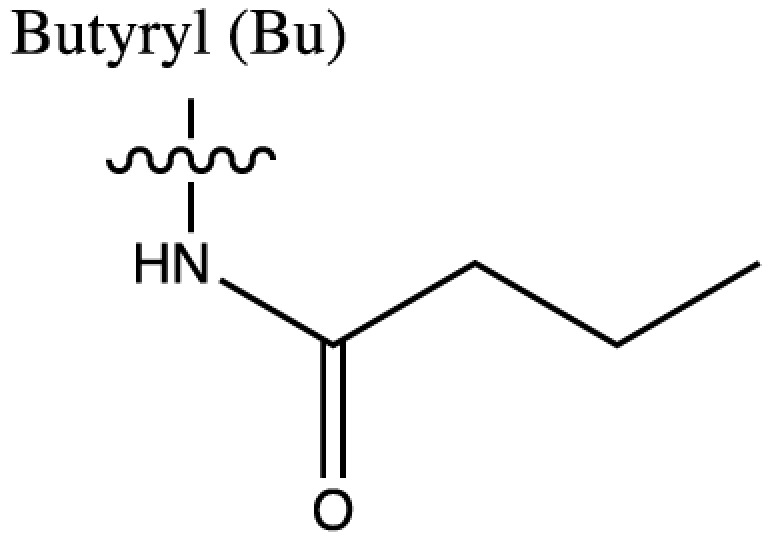	Hydrophobic	p300/CBP [78,93], PCAF [93], GCN5 [93,113]	SIRT1/2/3 [115]
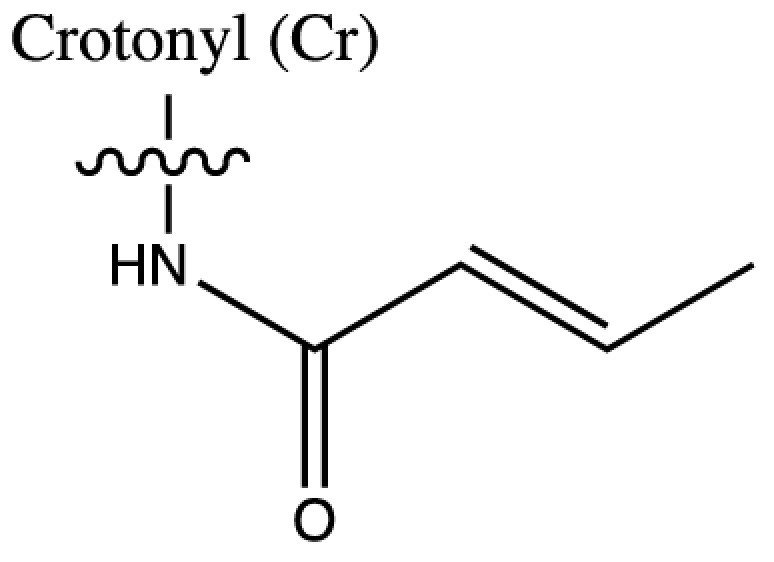	Hydrophobic	p300/CBP [10], MOF [95]	HDACs 1/2/3 [96,116–118], SIRT1/2/3 [119]
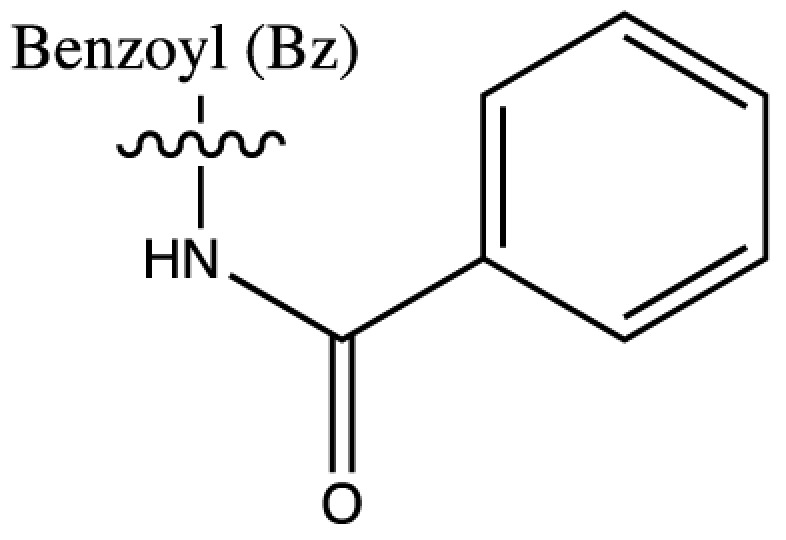	Hydrophobic		SIRT2 [83]
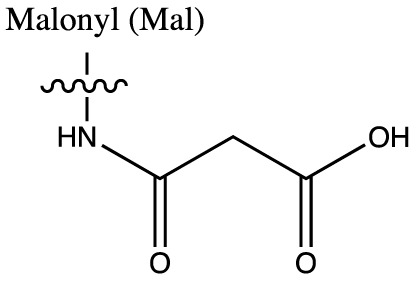	Acidic		SIRT5 [120]
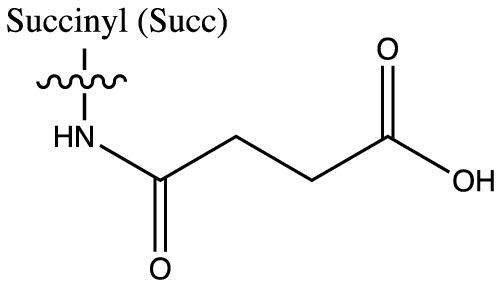	Acidic	GCN5 [86]	SIRT5 [120,121], SIRT7 [99]
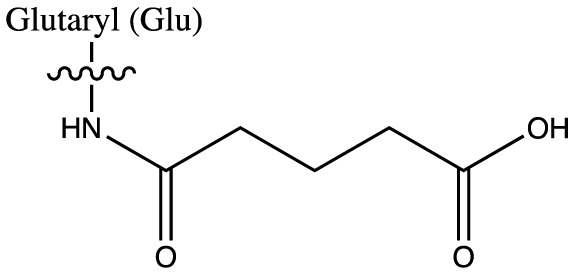	Acidic	p300 [81]	SIRT5 [81]
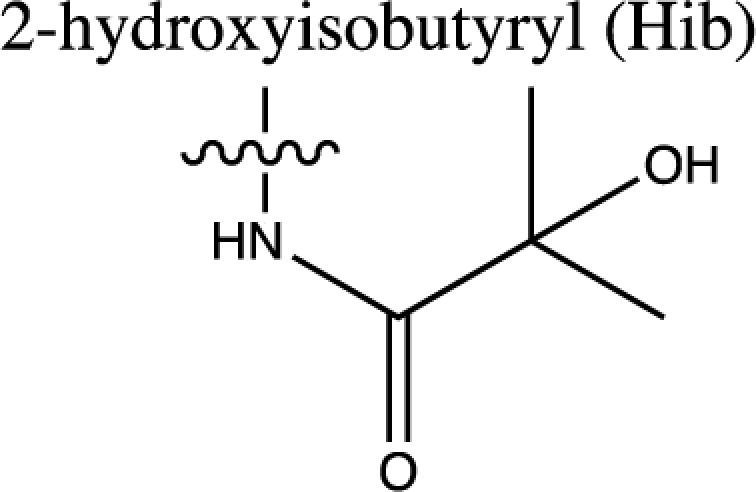	Polar	p300 [94], Tip60 [122]	HDACs 1/2/3 (largely 2 and 3) [80,122]
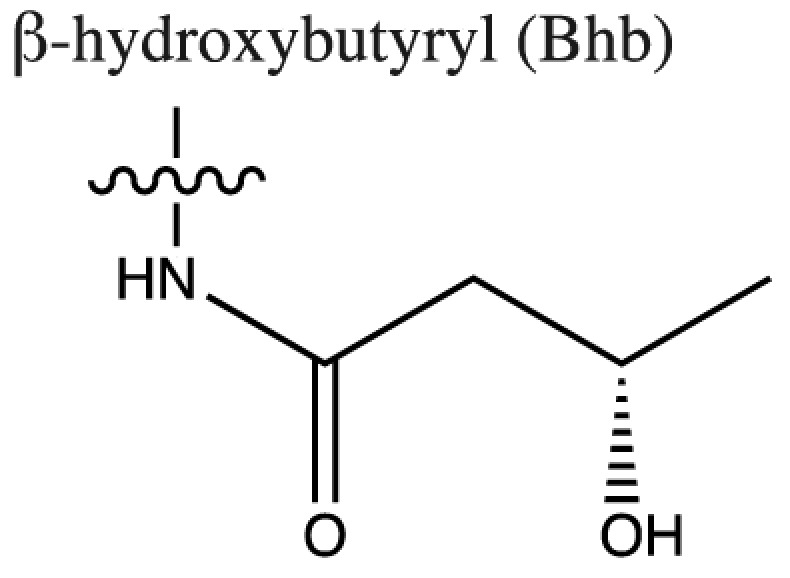	Polar	p300 [85]	

The chemical structures and properties of acyl groups and the enzymes which are currently known to catalyse the addition or removal of these from histones are shown.

A further point for debate is whether these modifications arise due to the chemical reactivity of acyl-CoAs without the need for enzymatic catalysis, as has been seen in the favourable conditions of the mitochondria (alkaline pH and high acyl-CoA concentrations) [88,89]. Recently, Simithy et al. [19] tried to address this and determined that all the histone-acyl modifications they studied could occur chemically (although the acyl-CoA concentrations used were far above physiological levels) as well as through HAT activity, albeit with decreased efficiencies for the acidic, branched or planar acyl-CoAs. Interestingly, the HATs tested showed a specificity for N-terminal histone tails whereas, sites of chemical addition were closer to the C-terminus [19]. The formation of high-energy cyclic anhydride intermediates that rapidly acylate proteins by succinyl-CoA and glutaryl-CoA suggests that K_Succ_ and K_Glu_ may occur non-enzymatically and perhaps this mechanism may be more prevalent for certain acylations than others [90]. Further studies are still required to determine the balance between chemical acylation and HAT activity. The levels of acyl-CoAs reflect the metabolic status of the cell [91] and studies have shown that altering acyl-CoA concentrations can modify the levels of histone acylations [10,19,92], highlighting an interesting link between cell metabolism and chromatin modifications. The relative abundance of each acyl-CoA and differences in the relative levels of these between cell types [19] may therefore, at least partially, regulate the abundance of different chromatin acylation marks.

## Physiological roles of diverse acylations

A number of studies have shown that similar to acetylation, many of the alternative acyl marks are found at transcriptionally active regions of the genome. For instance, H3K9_Bhb_ is enriched at gene promoters in mouse liver tissue [82], suggesting a role in transcriptional regulation. K_Cr_, K_Bu_, K_Pr_ and K_Hib_ have all been shown to directly stimulate transcription to a similar (or even greater) extent than acetylation, using cell-free assays [10,11,93,94]. As many of these marks occur at the same sites as acetylation, it raises the question as to whether their functions overlap or diverge. CBP/p300 mutants lacking acetyltransferase but retaining crotonyltransferase activity were still able to enhance transcription, suggesting a role for crotonylation in enhancing transcription [95]. Further studies indicate that crotonylation plays a role in maintaining the pluripotent state in mouse embryonic stem cells [96], assists histone replacement during spermatogenesis [87] and can reverse HIV latency [97], potentially providing a therapeutic opportunity. One recent study has suggested that malonylation of yeast H2A may lead to a chromosome segregation defect [98]. Hypersuccinylation, achieved by both the depletion of SIRT7 and succinate dehydrogenase, results in defects in DNA repair [99,100]. These studies highlight the ever-expanding functions of histone lysine acylations.

As discussed above in relation to K_Ac_, ‘readers’ of the histone modification play a critical role in interpreting and propagating the signal and the same appears to be the case for the newly identified acylations. Studies using known K_Ac_ reader proteins have identified a range of binding capabilities for the newer acyl marks. Human BDs have a general capability to bind K_Pr_ (as the hydrocarbon chain of K_Pr_ is only one carbon longer than K_Ac_) but not K_Cr_ or K_Bu_ (other than BRD9, CECR2 and TAF1) [101]. An investigation of acyl marks at H4K5/K8 showed that the first BD (BD1) of BRDT binds both H4K5_Ac_K8_Ac_ and H4K5_Ac_K8_Bu_; however, binding is abolished by butyrylation at H4K5 [11], suggesting a competition between acylations. It was subsequently shown that the binding of BRDT and BRD4-BD1 to H4K5 was enhanced by any acylation at H4K8 [102]. The Double PHD Finger (DPF) domains of two HATs MOZ and MORF have been shown to bind preferentially to H3K14_Cr_ and H3K14_Bu_ respectively [103,104]. The ability for these HATs to bind different acyl marks is thought to be vital for the spread of histone acylation which is proposed to help form and maintain open chromatin. In addition to BDs, proteins containing a YEATS domain, have shown a general preference for K_Cr_ over K_Ac_. The AF9 YEATS domain can bind to several K_Cr_ marks on histone H3 (K9, K18, K27) as well as K_Pr_ and K_Bu_ marks [105], while Taf14 binds preferentially to H3K9_Cr_ [106] and YEATS2 to H3K27_Cr_ [107]. Using a mutated version of TAF14 designed to selectively bind H3K9_Cr_ over H3K9_Ac_; Klein et al. [108] were able to show that there may be a differential requirement of H3K9_Ac_ and H3K9_Cr_ in the expression of TAF14-regulated genes. More recently it has been suggested that the YEATS domain of GAS41 is a pH-dependent reader of H3K122_Succ_ [109], hinting that there may be a further expansion of the reading capabilities of the YEATS domain family (reviewed in [110]). YEATS and PHD finger domains selecting for alternative acyl marks over acetylation is an exciting discovery, and though there is clearly far more to be discovered in terms of acyl readers, it may be the case that the recruitment of distinct readers by different acyl modifications leads to different transcriptional readouts.

## Summary

- Within the last decade, multiple histone acylation marks have been discovered that have distinct characteristics in addition to those of histone acetylation.
- These marks appear to be regulated by HATs and HDACs, but the contribution of non-enzymatic acylation cannot be discounted.
- There are clear links with the newer acylations and active transcription, which recruit diverse reader proteins that are able to further modify or remodel chromatin.
- There is clearly still a lot to discover in terms of the function and physiological relevance of these diverse chemical modifications and (no pun intended) this marks a very exciting time in the study of chromatin biology.

## Abbreviations

BD: bromodomain
eRNA: enhancer RNA
HAT: histone acetyltransferase
HDAC: histone deacetylase
K_Ac_: acetyl-lysine
K_Bhb_: β-hydroxybutyryl-lysine
K_Bu_: butyryl-lysine
K_Bz_: benzoyl-lysine
K_Cr_: crotonyl-lysine
K_Glu_: glutaryl-lysine
K_Hib_: 2-hydroxyisobutyryl-lysine
K_Mal_: malonyl-lysine
K_Pr_: propionyl-lysine
K_Succ_: succinyl-lysine
PHD: Plant homeodomain
PTM: Post-translational modification
TSS: transcription start site
